# Health Information Seeking Behavior on Social Networking Sites and Self-Treatment: Pilot Survey Study

**DOI:** 10.2196/51984

**Published:** 2023-12-20

**Authors:** Reginald A Silver, Chandrika Johnson

**Affiliations:** 1 Belk College of Business University of North Carolina at Charlotte Charlotte, NC United States; 2 Department of Health, Physical and Secondary Education Fayetteville State University Fayetteville, NC United States

**Keywords:** health care seeking behavior, online social networking, sociodemographic factors, community survey, logistic regression, self-treatment

## Abstract

**Background:**

Social networking site use and social network–based health information seeking behavior have proliferated to the point that the lines between seeking health information from credible social network–based sources and the decision to seek medical care or attempt to treat oneself have become blurred.

**Objective:**

We contribute to emerging research on health information seeking behavior by investigating demographic factors, social media use for health information seeking purposes, and the relationship between health information seeking and occurrences of self-treatment.

**Methods:**

Data were collected from an online survey in which participants were asked to describe sociodemographic factors about themselves, social media use patterns, perceptions about their motivations for health information seeking on social media platforms, and whether or not they attempted self-treatment after their social media–related health information seeking. We conducted a binomial logistic regression with self-treatment as a dichotomous categorical dependent variable.

**Results:**

Results indicate that significant predictors of self-treatment based on information obtained from social networking sites include race, exercise frequency, and degree of trust in the health-related information received.

**Conclusions:**

With an understanding of how sociodemographic factors might influence the decision to self-treat based on information obtained from social networking sites, health care providers can assist patients by educating them on credible social network–based sources of health information and discussing the importance of seeking medical advice from a health care provider.

## Introduction

Health information seeking behavior (HISB) has garnered much research attention during a time of nearly ubiquitous access to information. The digital landscape is continuously evolving, and people are constantly “plugged in.” The seemingly free-flowing availability of information from numerous digital platforms has begun to influence how people seek out health-related information and how this information is used in the context of subsequent health-related behaviors. While concrete definitions of HISB remain mixed, there is some research consensus that HISB can be understood as having two dimensions: one dimension is associated with the extent to which health information is sought and the other dimension is associated with the method by which the information is sought [1]. This research study aims to add to the existing body of knowledge concerned with this second dimension, the method by which HISB occurs. Extant research related to HISB tends to focus primarily on the use of the internet as the method of information seeking. In our context, we specifically explore the role that social networking platforms play in HISB.

Percheski and Hargittai [2] studied the HISB exhibited among college students and found that HISB was more likely to occur in younger women. This finding signals a recurring theme in HISB-related research as it concerns the factors that predict HISB. Wang et al [3] investigated social determinants of HISB and demonstrated that sociodemographic factors (ie, age, gender, education level, physical activity, smoking status, and income level) can explain the likelihood that an individual will engage in HISB. Some of these determinants are consistent even when the locale, the sample characteristics, and the information platforms are changed. The study by Wang et al [3] benefited from a relatively general population sample of adults in Hong Kong, and it explored a number of different platforms by which HISB was conducted, including television, radio, and the internet. Basch et al [4] conducted a study that sampled college students and found that HISB was more likely to be exhibited by students who identified as female and students who identified as non-White. The findings from Basch et al [4] are similar to the findings from Percheski and Hargittai [2], with both studies having focused on HISB among college students. Both Percheski and Hargittai [2] and Basch et al [4] highlight a greater likelihood of HISB occurring among younger women. These prior studies demonstrate the influence that demographic factors have on HISB.

Other research has been dedicated to understanding the motivations behind HISB. Motivation to engage in HISB has been attributed to the information seeker possessing a known health concern [5]. In some cases, health information seekers have sought health information on social networks prior to actually being seen by a health care provider [6]. HISB has been linked to the presence of chronic illnesses. Health information seekers with chronic illnesses, particularly those who have more than one chronic illness, tend to be more frequent users of the internet for health information seeking purposes [7]. In addition to these intrinsic motivations to seek health information, HISB is often encouraged by health care providers. Much has been researched in the way of promoting self-engagement in one’s own care. HISB has been promoted by health care providers as a means to facilitate patient engagement [8].

As more and more people have turned to social network–based resources, researchers have cautioned us about 2 salient issues with this type of HISB: the quality of the health-related information available on social networks and disparities in access to this information across different groups of people [9]. Because the level of expertise and credibility of some social network–based health information sources are highly subjective, there are mixed perceptions about the amount of trust that health information seekers put into the quality of the information that they find [10]. As it relates to disparate access to social network–based health information, information seekers who have limited internet access or limited literacy in the use of the internet are unable to access this health information in the same way as literate users with unlimited access to the internet. This digital divide and the resulting inequity across sociodemographic groups has been studied in a broad context as it relates to the internet [11-14]. Feng and Xie [15] have gone as far as to say that this digital divide also manifests in disparate access to online health information as a function of disparate access to social networking sites.

We incorporate what we have learned from extant research about HISB and aim this study at filling gaps in the existing body of knowledge for HISB. Specifically, we explore the sociodemographic determinants of HISB with the hope of identifying some new, previously undocumented determinant or alternatively, adding to the generalizability of previous researchers’ work by conducting our study with a relatively general population sample of health information seekers. The study also aims to create a better understanding of the influence that perceptions about chronic diseases and the trustworthiness of health information obtained from social networking sites have on a health-related outcome, in this case self-treatment. We also consider the role that provider trust may play in affecting how HISB may result in an occurrence of self-treatment. We position this study as a response to the call for additional research into how health information seeking might influence health management and to elucidate additional facets of HISB [16].

The study poses 4 research questions (RQs). RQ1: What are the sociodemographic factors that influence social networking site use for social network–based HISB? RQ2: Is health information seeking with social networking sites a function of perceived health condition or a function of trust in the information found? RQ3: What is the role of provider trust in the use of social networking sites for health information seeking? RQ4: Does health information seeking using social networking sites result in episodes of self-treatment?

## Methods

### Overview

A pilot survey was developed to capture responses from participants that would provide insight into sociodemographic factors, perceptions about social networking site use to obtain health-related information, perceptions about their own health status, trust in the health-related information received from social media platforms, level of trust in their health care provider, and whether or not they attempted self-treatment after seeking health-related information on social networking platforms.

### Ethical Considerations

The survey was reviewed by the institutional review board at the University of North Carolina at Charlotte (18-0521). Links to the survey were distributed electronically on social media platforms, including Facebook, Instagram, Twitter, Google+, and LinkedIn. The survey link was also distributed via direct email to contacts known by the authors. The survey link also included an informed consent form for respondents to acknowledge in order to proceed to the survey. Participation in the survey was completely optional, and responses were anonymized so that individuals who participated in the survey could not be identified.

### Sociodemographic Factors

The electronic survey captured 6 sociodemographic factors that were used to describe characteristics of the survey respondents. Each factor was measured as a categorical variable and subsequently dummy coded for the logistic regression. These factors were all treated as independent variables in the logistic regression model. Sociodemographic factors included race, age, gender, education level, health status, and exercise frequency.

### Race

Respondents were able to self-identify their race by choosing from 1 of 7 options. The categories for race included Native American, Hispanic, African American, White, multiracial, Asian, and other. For the purposes of the logistic regression, White was designated as the reference category to which all the other racial categories would be compared.

### Age

The age of each respondent was measured as 1 of 5 different age groups. The age groups that were provided for respondents to choose were 18-20 years, 20-30 years, 30-40 years, 40-50 years, and >50 years.

### Gender

Three options to record gender were provided. Respondents self-identified as either male, female, or other. The provision of only 3 options to record gender will be addressed later in the paper as a limitation of the study in fully capturing the expanding options that are available for individuals to self-identify their gender.

### Education

There were 6 options available for respondents to record the highest level of education that they had achieved. The options included “did not complete high school,” “diploma (high school),” “2-year degree,” “4-year degree,” “master’s degree,” and “doctoral degree.”

### Health Status

Health status was recorded as 1 of 5 options that allowed respondents to self-report whether they were in perfect health or had a chronic disease. This survey item was analyzed as the variable “HealthStatus” and identified whether a person reported being in perfect health, had 1 chronic illness, had 2 chronic illnesses, had 3 chronic illnesses, or had more than 3 chronic illnesses.

### Exercise Frequency

In an effort to understand the impact that exercise, identified here as a measure of health-seeking behavior, has on health information seeking, and ultimately the propensity to attempt self-treatment, respondents were asked to report the frequency with which they engaged in some form of exercise. Responses to this survey item were “never,” “once per week,” “2-3 times per week,” and “>3 times per week.”

### Provider Trust

To obtain perceptions about the level of provider trust among the survey respondents, the survey included the item “Describe your level of trust in your health care provider.” Responses were measured with a 3-point Likert scale. The response options were 1=“I do not trust what my health care provider tells me,” 2=“I somewhat trust what my health care provider tells me,” and 3=“I trust everything that my health care provider tells me.”

### Social Network Health Information Impact

A survey item was included to assess perceptions about the potential impact that health information received from social networking sites might have on actual health seeking behavior. Respondents were asked, “To what degree does information from social media sites affect your health seeking behavior?” Response options were 1=“not much,” 2=“somewhat,” and 3=“very much.” The item was intended to capture the level at which respondents incorporate health information received from social networking sites into their self-treatment.

### Social Network Health Information Trust

To explore respondent perceptions about the level of trust that they had in health-related information obtained from social networking sites, respondents were asked, “Describe the level of trust that you have in health-related information that you obtain from social media sites.” Response options were 1=“I do not trust the information,” 2=“I somewhat trust the information,” and 3=“I trust the information.”

### Perceived Susceptibility

Perceived susceptibility represents the level to which a person believes that they might be at risk of contracting a particular health condition [17]. Respondents were asked to indicate their self-perception of the likelihood that they would contract a serious condition by responding to the following survey item: “Using the scale below, rate how susceptible you think you are to disease.” Response options were chosen from a 7-point Likert scale with the terminal values of 1=“very strongly disagree” to 7=“very strongly agree.”

### Improvement in Health Status Because of Social Network Health Information

Respondents were asked explicitly if they believed that they had improved their health status because of information that they obtained while seeking health-related information on social networking sites. The survey item was presented as, “I believe that I have improved my health because of information that I have found on social media.” Available response options for this survey item were presented on a 7-point Likert scale with terminal values of 1=“very strongly disagree” and 7=“very strongly agree.”

### Self-Treatment

Self-treatment as a function of assimilating health information obtained from social networking sites was recorded as a binary categorical variable. The survey item was presented as “Do you try to treat yourself after obtaining health information from social media?” Respondents selected 1 for “yes” and 0 for “no.” This application of the dependent variable as a dichotomous measure lends itself to the use of logistic regression as a method of analysis [18]. The logistic regression was conducted in RStudio (Posit Software).

## Results

Data were obtained from 166 respondents. After eliminating 28 incomplete surveys, another 31 surveys were eliminated from the study because of missing values. The elimination of incomplete surveys and surveys with missing values resulted in a sample size of n=107. The sample of survey respondents represents a cross-section of social network–based health information seekers in the United States. Representation of the survey respondents by race was skewed, however, due to the majority of the respondents identifying as predominantly African American or White. The race of the survey respondents was nearly evenly distributed between African American (n=44, 41.1%) and White (n=50, 46.7%) individuals, with smaller percentage distributions for the other racial groups that were represented (Table 1).

**Table 1:** Sociodemographic characteristics of survey respondents (n=107).

Characteristics		Respondents, n (%)
**Race**		
	African American	44 (41.1)
	Asian	3 (2.8)
	Hispanic	3 (2.8)
	Multiracial	4 (3.7)
	Native American	2 (1.9)
	Other	1 (0.9)
	White	50 (46.7)
**Age group (years)**		
	>50	14 (13.1)
	18-20	7 (6.5)
	20-30	38 (35.5)
	30-40	21 (19.6)
	40-50	27 (25.2)
**Gender**		
	Female	77 (71.9)
	Male	29 (27.1)
	Other	1 (0.9)
**Education**		
	Diploma (high school)	16 (14.9)
	2-year degree	15 (14)
	4-year degree	38 (35.5)
	Master’s degree	31 (29)
	Doctoral degree	7 (6.5)
**Health status**		
	1 chronic illness	25 (23.4)
	2 chronic illnesses	8 (7.5)
	3 chronic illnesses	2 (1.9)
	>3 chronic illnesses	1 (0.9)
	Perfectly healthy	71 (66.4)
**Exercise frequency**		
	>3 times per week	22 (20.6)
	2-3 times per week	34 (31.8)
	Once per week	27 (25.2)
	Never	24 (22.4)

Respondents were primarily aged between 20-30 years (n=38, 35.5%). The second largest age group represented in the respondent sample was the group of respondents who identified as being aged between 40-50 years (n=27, 25.2%). Respondents identified predominantly as female (n=77, 71.9%). The remaining survey respondents identified as male (n=29, 27.1%) or other (n=1, 0.9%). The majority of survey respondents had obtained a degree from a 4-year institution (n=38, 35.5%). Most respondents indicated that they were in perfect health (n=71, 66.4%). Frequency of exercise was reported as occurring most often between 2 to 3 times per week.

Most respondents (n=62, 58%) indicated that they somewhat trust the health-related information that they receive from their health care provider (Table 2). When asked whether they thought that the health-related information that they found on social networking sites was impactful to their health-seeking behavior, most respondents (n=58, 54.2%) selected the option “not much.”

Most respondents (n=75, 70.1%) “somewhat trust” the health-related information that they obtain from social networking sites. When asked if they felt that they were susceptible to disease, respondents provided mixed responses with a nearly even split between “neutral” (n=32, 29.9%) and “agree” (n=33, 30.8%). The majority of respondents (n=43, 40.2%) were “neutral” in their responses about whether the health information that they obtained on social networking sites actually helped them improve their health status.

Episodes of self-treatment after social network–based health information seeking were reported by a minority of survey respondents (n=41, 38.3%). The majority of respondents reported that they did not attempt to treat themselves after obtaining health information from social networking sites (Table 3).

**Table 2:** Distribution of responses (n=107).

Responses			Respondents, n (%)
**Provider trust**			
	Do not trust information from provider	2 (1.9)	
	Somewhat trust information from provider	62 (58)	
	Trust information from provider	43 (40.2)	
**Impact of information**			
	Not much	58 (54.2)	
	Somewhat	40 (37.4)	
	Very much	9 (8.4)	
**Trust in social network health information**			
	Do not trust the information	26 (24.3)	
	Somewhat trust the information	75 (70.1)	
	Trust the information	6 (5.6)	
**Perceived susceptibility**			
	Very strongly disagree	4 (3.7)	
	Strongly disagree	16 (15)	
	Disagree	16 (15)	
	Neutral	32 (29.9)	
	Agree	33 (30.8)	
	Strongly agree	5 (4.7)	
	Very strongly agree	1 (0.9)	
**Improved health status**			
	Very strongly disagree	10 (9.4)	
	Strongly disagree	5 (4.7)	
	Disagree	13 (12.2)	
	Neutral	43 (40.2)	
	Agree	25 (23.4)	
	Strongly agree	9 (8.4)	
	Very strongly agree	2 (1.2)	

**Table 3:** Self-treatment among respondents (n=107).

Responses			Respondents, n (%)
**Self-treatment**			
	No	66 (61.7)	
	Yes	41 (38.3)	

Although the majority of survey respondents reported that they did not attempt self-treatment after seeking health information from social networking sites, a moderately accurate predictive model was able to be defined. Assessing the number of “true positives” (the number of actual and predicted respondents who admitted self-treatment) along with the number of “true negatives” (the number of actual and predicted respondents who did not self-treat) resulted in an overall model accuracy of 78.5% and a sensitivity rate of 70.73% (Table 4).

Prior to conducting the logistic regression, we assessed our variables of interest to determine if there were any significant interitem correlations. We include the correlation matrix in Multimedia Appendix 1. We noted significant correlations between ExerciseFreq_3 and ExerciseFreq_2 (*r*=–.396; *P*<.001). ExerciseFeq_4 was significantly correlated with both ExerciseFreq_2 (*r*=–.296; *P*=.002) and ExcerciseFreq_3 (*r*=–.347; *P*<.001). Depending on the frequency at which respondents indicated that they exercised, they were less likely to exercise in a lower frequency category. SMITrust2 was significantly correlated with ExerciseFreq_2 (*r*=.191; *P*=.048). For respondents who indicated that they “somewhat trust” the information that they found on social media networks, they were also likely to indicate that they exercised approximately one time per week. SMITrust3 was significantly correlated with SMITrust2 (*r*=–.373; *P*<.001), indicating that if a respondent indicated that they “trust the information” that they found on social media network sites, they were less likely to respond that they “somewhat trust” the information that they found on social networking sites.

The results of the binomial logistic regression revealed that significant predictors of self-treatment after health information seeking on social networking sites include the level of trust in the information obtained from the social networking site, race (African American), and exercise frequency (Table 5).

Trust in the health-related information obtained from social networking sites was represented by the variables SMITrust1=“do not trust the information,” SMITrust2=“somewhat trust the information,” and SMITrust3=“trust the information.” Of these trust variables, only SMITrust2 (β=1.8695; *P*=.01) and SMITrust3 (β=3.7162; *P*=.02) were statistically significant. Respondents who “somewhat trust” the health information they obtain from social networking sites were 6.5 times (odds ratio [OR] 6.4849, 95% CI 1.66-3.52E+01) more likely to self-treat as compared to the reference group of individuals who did not trust the information on social networking sites. Individuals who “trust” the health information that they obtain from social networking sites were nearly 41 times (OR 41.1090, 95% CI 2.78-1.51E+03) more likely to self-treat than those who did not trust health information from social networking sites.

Race significantly predicted self-treatment but only among respondents who identified as African American (β=1.4413; *P*=.005). African Americans were 4 times (OR 4.2261, 95% CI 1.59-1.20E+01) more likely than their White counterparts to self-treat after obtaining health-related information from social networking sites.

Frequency of exercise significantly predicted self-treatment among respondents who stated that they exercised more than 3 times per week (β=-2.5437; *P*=.005). We note the negative coefficient for exercising more than 3 times per week and interpret this inverse relationship between frequent exercise and self-treatment. This finding suggests that respondents who more frequently engage in some form of exercise are actually less likely to self-treat as a result of health information seeking with social networking sites. While the odds ratio for this group of respondents was small (OR 0.0786, 95% CI .011-4.11E-01), the negative association between exercising more than 3 times per week and self-treatment is still statistically significant.

In terms of goodness-of-fit, the final model demonstrated a statistically significant improvement over the null model. Results of the log likelihood ratio test were as follows: Δ_df_=–11; ΔLogLikelihood=–21.756; χ^2^_11_=43.513 (*P*<.001). The final model resulted in a pseudo *R*^2^ of 0.3055. Because of the nature of logistic regression, we are cautious about stating that the model explains 31% of the variance in self-treatment. The pseudo *R*^2^ reported here is based on the McFadden *R*^2^. The same model produced a pseudo *R*^2^ of 0.331432 according to the Cox and Snell method and a pseudo *R*^2^ of 0.454083 according to the Nagelkerke method.

Sociodemographic factors identified in this study as predictors of social network–based health information seeking include trust in the information obtained from the social networking site, race, and exercise frequency. Health information seeking on social networking sites appears to be motivated by trust in the health information more than the criticality of the current health condition. We included provider trust as a variable in our logistic regression models, but provider trust did not significantly predict self-treatment as a result of social network–based HISB. Our final regression model supports a significant association between self-treatment and social network–based health information seeking.

To compare the results of our logistic regression to other methods of analysis, we conducted a number of post hoc tests. These post hoc tests serve as a robustness check of our findings. For the relationship between race and self-treatment, we conducted a chi-square test. The chi-square test supported a significant relationship between identifying as African American and identifying as someone who sought self-treatment (χ^2^_1,107_=10.822; *P*<.001). Similar chi-square tests were done for exercise frequency and social media information trust. For social media information trust, SMITrust2 (χ^2^_1,107_=5.450; *P*=.02), and for exercise frequency, ExerciseFreq >3 times per week (χ^2^_1,107_=7.138; *P*=.008) were found to be significantly associated with self-treatment. We observed similar results when we applied the Fisher exact test with each of the independent variables of race, exercise frequency, and social media information trust. The results of the Fisher exact tests were all significant (*P*=.001) indicating a significant association between the independent variables and the dichotomous dependent variable, self-treatment.

**Table 4:** Classification table. Accuracy: (29 + 55) / 107 = 0.7850; sensitivity: 29 / (29 + 12) = 0.7073

	Predicted self-treatment	Predicted absence of self-treatment
Actual self-treatment (n=41)	29	12
Actual absence of self-treatment (n=66)	11	55
Total (n=107)	40	67

**Table 5:** Factors that influence self-treatment after social network–based health information seeking.

		β	SE	*z*	*P* value	Odds ratio (95% CI^a^)
(Intercept)		–1.7536	0.8001	–2.192	.03	0.1731 (0.030-7.42E-01)
SMITrust2		1.8695	0.7603	2.459	.01	6.4849 (1.66-3.52E+01)
SMITrust3		3.7162	1.5485	2.4	.02	41.1090 (2.78-1.51E+03)
**Race**						
	African American	1.4413	0.5125	2.812	.005	4.2261 (1.59-1.20E+01)
	Asian	–16.3986	2161.7013	–0.008	.99	0.0000 (N/A^b^-7.18E+114)
	Hispanic	–16.4948	2027.1618	–0.008	.99	0.0000 (N/A-1.33E+107)
	Multiracial	2.2025	1.3613	1.618	.11	9.0472 (0.760-2.41E+02)
	Native American	–16.4862	2162.8038	–0.008	.99	0.0000 (N/A-3.03E+1.39)
	Other	–15.8124	3956.1804	–0.004	.99	0.0000 (N/A-inf^c^)
**ExerciseFreq**						
	>3 times per week	–2.5437	0.899	–2.83	.005	0.0786 (0.011-4.11E-01)
	2-3 times per week	–1.177	0.7094	–1.659	.09	0.3082 (0.072-1.20E+00)
	Once per week	–0.6136	0.7327	–0.837	.40	0.5414 (0.122-2.23E+00)

^a^Some CIs were incalculable because of how the software used treats dummy-coded variables for race.

^b^N/A: not applicable.

^c^inf: infinite.

## Discussion

While health information seeking theory remains fragmented, we have identified that sociodemographic factors can at least partially predict self-treatment as a result of health information seeking on social networking sites. Our findings highlight the need for caution as it relates to the quality of health information that is made available on social networking sites. Providers can capitalize on the level of trust that health information seekers place in the information that they find on social networking sites by developing a web presence for their medical practices on social media networking sites. Our results demonstrate that when health information seekers trust or even somewhat trust the information they obtain on social networking sites, self-treatment is more likely to occur. Whether this self-treatment is efficacious lies beyond the scope of this research study.

Our research also emphasizes the need to be concerned with the use of social networking sites for health information seeking purposes among the African American community. Our results suggest that African Americans are more likely to self-treat after obtaining health-related information from social networking sites. This assumes that the health information seeker has successfully navigated the digital divide that historically has been an obstacle in the African American community [19-23].

Within our pilot study, we note a number of limitations. Among these limitations is the unbalanced representation of different racial groups. Our study results were based on responses from people who identified predominantly as White or African American. A more comprehensive study should include a broader sampling strategy with more equitable representation between racial groups. Similarly, gender was captured in a predominantly binomial fashion. Future research on health information seeking should consider multiple gender classifications in an effort to understand more nuanced HISB. A more comprehensive study should also attempt to achieve a much larger sample size to improve the generalizability of the findings that we report. An additional complicating factor that must be considered for future research on health information seeking is the likely emergence of a propensity for individuals to increase their use of social networking sites as a source of health-related information in a postpandemic era, wherein attempts at self-treatment might increase as a direct result of fear of contracting COVID-19 as a result of seeing a provider.

## Appendix

Multimedia Appendix 1 Correlation matrix.

## Abbreviations

HISB: health information seeking behavior
OR: odds ratio
RQ: research question
