# Supplementary material for: Health Information Seeking Behavior on Social Networking Sites and Self-Treatment: Pilot Survey Study
Source: Online J Public Health Inform. 2023 Dec 20;15:e51984. doi: 10.2196/51984 (PMC10765284; doi:10.2196/51984)
Supplement: Multimedia Appendix 1 [file ojphi_v15i1e51984_app1.docx]

Appendix A

Correlation Matrix

|  |  | 1 | 2 | 3 | 4 | 5 | 6 | 7 | 8 | 9 | 10 |
| --- | --- | --- | --- | --- | --- | --- | --- | --- | --- | --- | --- |
| 1 | Native_American |  |  |  |  |  |  |  |  |  |  |
| 2 | Hispanic | -0.023 |  |  |  |  |  |  |  |  |  |
| 3 | African_American | -0.115 | -0.142 |  |  |  |  |  |  |  |  |
| 4 | Multiracial | -0.027 | -0.033 | -0.165 |  |  |  |  |  |  |  |
| 5 | Asian | -0.023 | -0.029 | -0.142 | -0.033 |  |  |  |  |  |  |
| 6 | Other | -0.013 | -0.016 | -0.081 | -0.019 | -0.016 |  |  |  |  |  |
| 7 | ExerciseFreq_2 | -0.080 | 0.032 | 0.039 | -0.001 | 0.032 | -0.056 |  |  |  |  |
| 8 | ExerciseFreq_3 | -0.094 | -0.116 | -0.040 | 0.077 | 0.006 | -0.066 | -0.396** |  |  |  |
| 9 | ExerciseFreq_4 | 0.101 | 0.054 | -0.049 | 0.022 | 0.054 | -0.049 | -0.296** | -0.347** |  |  |
| 10 | SMITrust2 | -0.061 | -0.013 | 0.131 | 0.021 | 0.111 | -0.149 | 0.191* | 0.051 | -0.072 |  |
| 11 | SMITrust3 | -0.034 | -0.041 | -0.039 | 0.166 | -0.041 | -0.024 | -0.142 | -0.079 | -0.023 | -0.373** |
